# Cell-Penetrating Antimicrobial Peptides Derived from an Atypical Staphylococcal δ-Toxin

**DOI:** 10.1128/spectrum.01584-21

**Published:** 2021-12-22

**Authors:** Kathyana Deeyagahage, Antonio Ruzzini

**Affiliations:** a Department of Veterinary Microbiology, University of Saskatchewan, Saskatchewan, Canada; b Department of Biochemistry, Microbiology and Immunology, University of Saskatchewan, Saskatchewan, Canada; Emory University School of Medicine

**Keywords:** MRSA, antimicrobial peptides, bactericidal activity, delta-toxin, phenol-soluble modulins, staphylococci

## Abstract

Revisiting underutilized classes of antibiotics is a pragmatic approach to the identification of alternative therapies for antimicrobial-resistant pathogens. To this end, we designed and screened a set of seven staphylococcal δ-toxin-inspired peptides (STIPs) for antibacterial activity against methicillin-resistant Staphylococcus aureus (MRSA). Furthermore, a pathogen-specific protease was leveraged to generate shorter peptides from these δ-toxin derivatives to expand the screen of putative antimicrobial peptides (AMPs) and to counterscreen against AMP inactivation. Remarkably, a 17-amino acid peptide based on the atypical δ-toxin sequence of Staphylococcus auricularis was discovered to possess an ability to kill MRSA and related pathogens. An alanine scan and series of rational substitutions improved AMP activity, and phenotypic assays characterized the STIPs’ ability to rapidly interact with and permeabilize the staphylococcal membrane without causing lysis on a commensurate timescale. Instead of rapid lysis, both l- and d-enantiomers of STIP3-29, an AMP with low micromolar activity, were observed to penetrate and accumulate within cells. Finally, we observed that STIP3-29 was capable of controlling MRSA infection in a three-dimensional skin infection model. Overall, the results suggest that this unconventional source of AMPs can provide promising candidates for further development as therapeutic agents.

**IMPORTANCE** The continued emergence and global distribution of infections caused by antimicrobial-resistant pathogens fuel our perpetual need for new or alternative therapies. Here, we present the discovery and initial characterization of bacterial cell-penetrating AMPs that were based on a family of virulence factors. In contrast to the multitude of AMPs that are sourced from animals, these potential therapeutic molecules have not undergone extensive selection for their antimicrobial properties and have proven to be amenable to activity-optimizing modifications. The staphylococcal toxin-inspired peptides described here represent a source of AMPs that can kill common opportunistic pathogens, such as MRSA, and have the potential to be improved for application in medicine.

## INTRODUCTION

Opportunistic infections caused by Staphylococcus aureus challenge the well-being of individuals and health care systems (1). The prevalence of staphylococcal infections reflects high rates of carriage, and the severity of disease ranges from mild skin inflammation to fatal septicemia (2). The use of antimicrobials to treat infections has selected for the global emergence of strains that encode resistance to β-lactams that are collectively named methicillin-resistant S. aureus (MRSA) (3). Accordingly, there is an urgent need to discover new therapies to prevent and treat disease caused by S. aureus.

Antimicrobial peptides (AMPs) are promising candidates for the treatment of skin and soft tissue infections caused by MRSA. Their biophysical properties and the fact that many have been naturally selected to play protective roles at animal-microbe interfaces suggest that AMPs are best suited for topical applications. The majority of AMPs under preclinical and clinical evaluation are based on animal host defense peptides (HDPs) (4–6). For example, the human cathelicidin LL-37 is a short cationic α-helical HDP that has been the subject of extensive structure-activity relationship studies and rational design experiments (7). A series of engineering efforts have resulted in peptides that are more potent antimicrobials than LL-37: 17BIPHE2 and SAAP-148 are preeminent among recent work to develop cathelicidin-inspired drugs (8, 9).

Bacteria produce short α-helical peptides reminiscent of the cathelicidins. The phenol-soluble modulins (PSMs) are staphylococcal peptides that not only resemble the cathelicidins but have also been demonstrated to colocalize and cooperate with HDPs to kill bacteria *in vitro* and in a murine infection model (10, 11). While members of this genus-specific peptide family are known as cytotoxic virulence factors with surfactant properties that facilitate biofilm structuring and remodeling (12, 13), a few PSMs or derivatives thereof have antibacterial properties (10, 11, 14–23). The antibiotic moonlighting activities and the antibacterial mechanism of PSM action, however, are poorly characterized.

Staphylococcal δ-toxins are members of the PSM peptide family. There are hundreds of PSM sequences encoded by bacteria within the genus *Staphylococcus*, including the short PSMα and longer PSMβ subtypes. The δ-toxins, which are considered members of the α-subtype, were well-known prior to the discovery of the broader PSM family. They are characterized as short ∼25 amino acid peptides with cationic residues concentrated near their C terminus. While there has been significant attention given to the more recently discovered PSMs, the δ-toxins are the most diverse of α-subgroup. There are >50 unique δ-toxins that have been reported in public nucleotide sequence databases that share as low as 36% sequence identity. The other PSMα sequences are generally better conserved and more hydrophobic than the δ-toxins, and while both derive cationic character from lysine residues, the δ-toxins also possess regularly spaced acidic amino acids. The functional consequences of the natural sequence diversity of the PSMs are unknown, though substitutions to relatively short peptides are suspected to affect one or more of the biological activities. Accordingly, we conducted a proof-of-concept study to demonstrate the varied capacity of δ-toxin-inspired peptides to act as AMPs. We report the discovery, characterization, and optimization of staphylococcal toxin-inspired peptides (STIPs) with cell-penetrating bactericidal activity.

## RESULTS AND DISCUSSION

### An uncommon δ-toxin sequence encodes antimicrobial activity.

**FIG 1:**
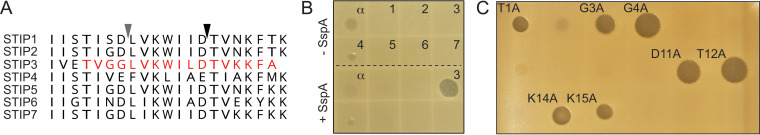
(A) Primary sequences of STIPs with arrows indicating common SspA cleavage sites. (B) A spot-on-lawn assay showing the effect of STIPs on S. aureus growth. Spots below the dashed line are of proteolytic reaction mixtures. (C) A spot-on-lawn assay showing the antibiotic activity of single-site alanine variants of STIP3-1 (red in panel A).

We based our collection of putative AMPs on 7 δ-toxins from 6 *Staphylococcus* spp. that are members of the human microbiome. The cultivation-independent approach, which relied on synthetic peptides, allowed for the inclusion of potential AMPs from well-characterized (e.g., S. aureus and S. epidermidis), infrequently studied (*S. auricularis*, *S. cornubiensis*, and *S. massiliensis*), and anaerobic (*S. saccharolyticus*) taxa. The δ-toxins were specifically selected among the short α-type PSMs since they possess regularly spaced acidic amino acids. The presence of these amino acids makes the δ-toxins susceptible to cleavage by the staphylococcal secreted protease SspA. In turn, this enzyme was used to counterscreen for the ability of S. aureus to destroy potential AMPs. Moreover, SspA was used *in vitro* to generate a larger library of smaller putative bioactive peptides from the initial set of 7 staphylococcal δ-toxin-inspired peptides (STIPs) (Fig. 1A; Table S1). Proteolysis was expected to release C-terminal fragments with similarities to the cationic hexa- and heptapeptides derived from PSMα3, (K)LFKFFK, that showed modest inhibition of Micrococcus luteus and S. hominis (21). In anticipation of SspA-mediated cleavage, N-terminal residues preceding the first conserved acidic amino were omitted from the STIPs. Overall, the 7 putative AMPs sequences share between 38 and 95% identity, have predicted isoelectric points between 8.4 and 10.0, possess at least two acidic amino acids, and begin with a pair of hydrophobic residues. The latter characteristic resembles the S. aureus PSMα1Δ1–2 and PSMα2Δ1–2 peptides, which possess an antibiotic activity that was once misattributed to a staphylococcal lantibiotic (15).

We employed spot-on-lawn assays to screen the STIPs as antibiotics. Crude peptides (50 μg, ≥70% purity; Table S1) were deposited on agar surfaces inoculated with either M. luteus ATCC 4698 or S. aureus ATCC 29213. M. luteus was inhibited by 5 STIPs and the aforementioned PSMα1Δ1–2 (Fig. S1). None of the STIPs prevented S. aureus growth; however, SspA-treated STIP3 was unique in showing antibacterial activity (Fig. 1B). To identify active and inactive components of the SspA:STIP3 reaction mixture, 4 possible SspA-generated STIP3 products were screened as individual and peptide mixtures (Fig. S2). A 17-amino acid peptide, named STIP3-1 (Fig. 1A, red), was assigned as the major antimicrobial component of the reaction mixture. Notably, STIP3-1 is characterized by a fortuitously missed SspA cleavage after Asp11, and subsequent SspA digestion experiments confirmed that further proteolysis can occur (Table S3). The STIP3 sequence, which was based on the δ-toxin of *S. auricularis*, a resident of the human ear, possesses an atypical distribution of acidic amino acids. Sequence comparison of 52 unique δ-toxin sequences revealed that this irregular distribution occurs in only 2 of the other 51 unique sequences (Fig. S3), highlighting the value of investigating δ-toxin sequence diversity.

Little is known about the action of SspA on the δ-toxins, but both are produced during infection (12, 24), and deformylated PSMs were reported as the major constituents of S. aureus amyloid fibers (25). We observed that the deformylated *S. auricularis* δ-toxin and STIPs were susceptible to SspA cleavage *in vitro* but that formylation protected the full-length δ-toxin against proteolysis (Table S3). Although these experiments cannot inform on the natural occurrence of δ-toxin-derived AMPs, they demonstrate the value of expanding AMP libraries via selected and limited proteolysis.

### Optimization of STIPs through rational design.

**FIG 2:**
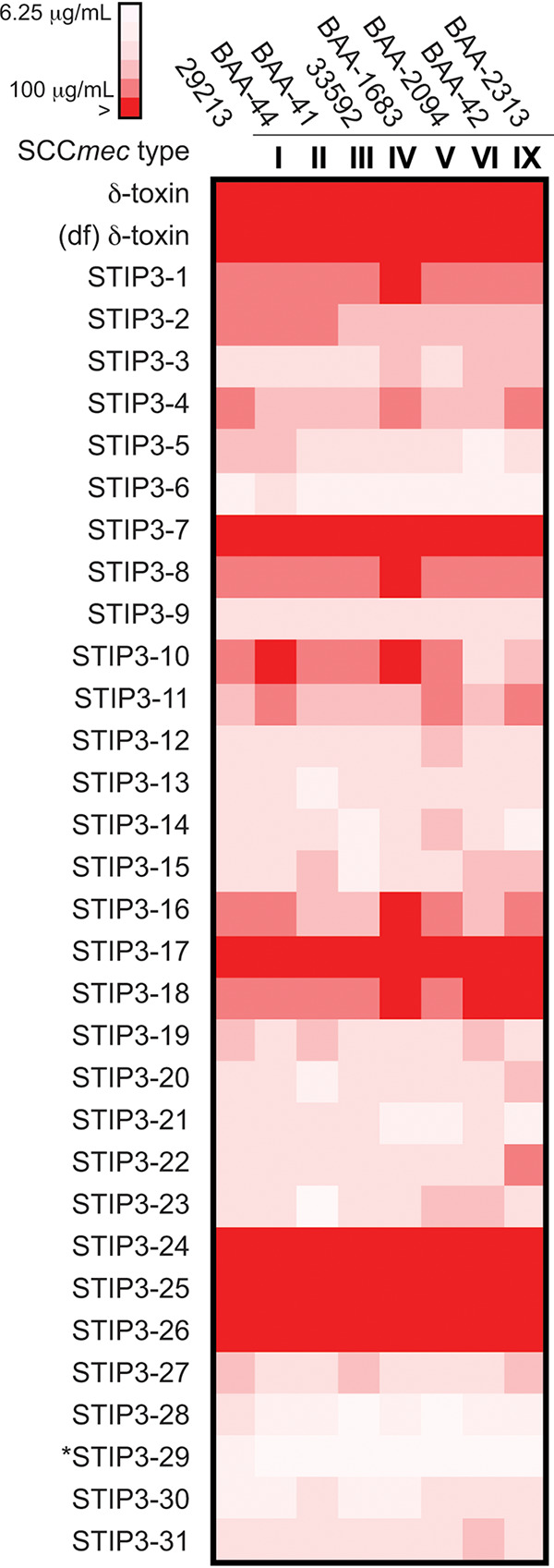
Heatmap illustrating the activities of STIP3 variants, the *S. auricularis* δ-toxin, and its deformylated (df) form. The > symbol indicates that no inhibition was observed at 100 μg/mL for the STIP3 variants or 200 μg/mL for the δ-toxins. The most potent AMP, STIP3-29, is indicated by an asterisk.

In order to assess and improve AMP activity, we performed an alanine scan to identify sequence determinants thereof. All single-site alanine-substituted peptides retained their ability to inhibit M. luteus (Table S4). Seven of the 16 STIP3-1 variants inhibited S. aureus growth on a solid agar surface (Fig. 1C), whereas T1A, D11A, T12A, and V13A maintained activity in nutrient broth at 200 μg/mL (Table S4). These results and a report that the central α-helical core of the S. aureus δ-toxin could be converted into an AMP through the inclusion of additional lysine residues (22) guided the design of 30 STIP3-1 variants (STIP3-2 to STIP3-31; Table S5). This collection was comprised of 10 single-site variants, including substitutions to eliminate the fortuitously missed Asp11 cleavage site, with the remaining 20 peptides differing at multiple sites to increase overall or local cationic charges or to have altered hydrophobicity. We evaluated the ability of these STIPs to inhibit methicillin-sensitive S. aureus ATCC 29213 and a panel of seven MRSA strains defined by distinct SCC*mec* genotypes (Fig. 2; ATCC MP-2). As expected, the incorporation of regularly spaced basic residues, including lysine, arginine, and ornithine, resulted in 4- to 8-fold improvements to STIP variant activity. A combination of these substitutions with hydrophobic residues or the inclusion of additional hydrophobic residues, resulted in peptides that no longer inhibited S. aureus. A 16-fold improvement to antibiotic activity was observed in the most potent peptide in the collection, STIP3-29, which differed from STIP3-1 in that Asp11 was replaced by a lysine and the C terminus was amidated. STIP3-29 inhibited MRSA at 6.25 μg/mL (3.3 μM; Table 1) and M. luteus at 1.56 μg/mL (850 nM). Further evaluation of STIP3-29 against a series of additional *Staphylococcus* spp., including clinical S. pseudintermedius isolates that caused canine and human infection (26), revealed MICs within the aforementioned concentration range (Table S6). In contrast, the bioactivity of STIP3-29 against a small panel of *Gammaproteobacteria* was variable: Klebsiella pneumoniae and Pasteurella multocida were resistant whereas Pseudomonas aeruginosa, Escherichia coli, and Mannheimia haemolytica were sensitive to the AMP at 50, 12.5, and 6.25 μg/mL, respectively (Table S6). Overall, the *in vitro* anti-staphylococcal activity of STIP3-29 is comparable to that reported for many AMPs based on animal HDPs.

**TABLE 1:** STIP3 variant sequences, helicity, and MICs for MRSA ATCC 33592

Peptide ID	Sequence or modification(s)	α-Helical content (%)	MIC (μg/mL)
STIP3-1	TVGGLVKWILDTVKKFA	67	100
STIP3-29	TVGGLVKWILKTVKKFA-NH2	61	6.25
d-STIP3-29	All d-enantiomer	47	6.25
STIP3-29-D1	d-K11	51	25
STIP3-29-D3	d-K7, d-K11, d-K15	32	>100
FAM-l-STIP3-29	N-terminal FAM	72	25
FAM-d-STIP3-29	N-terminal FAM, all d-enantiomers	55	100

**FIG 3:**
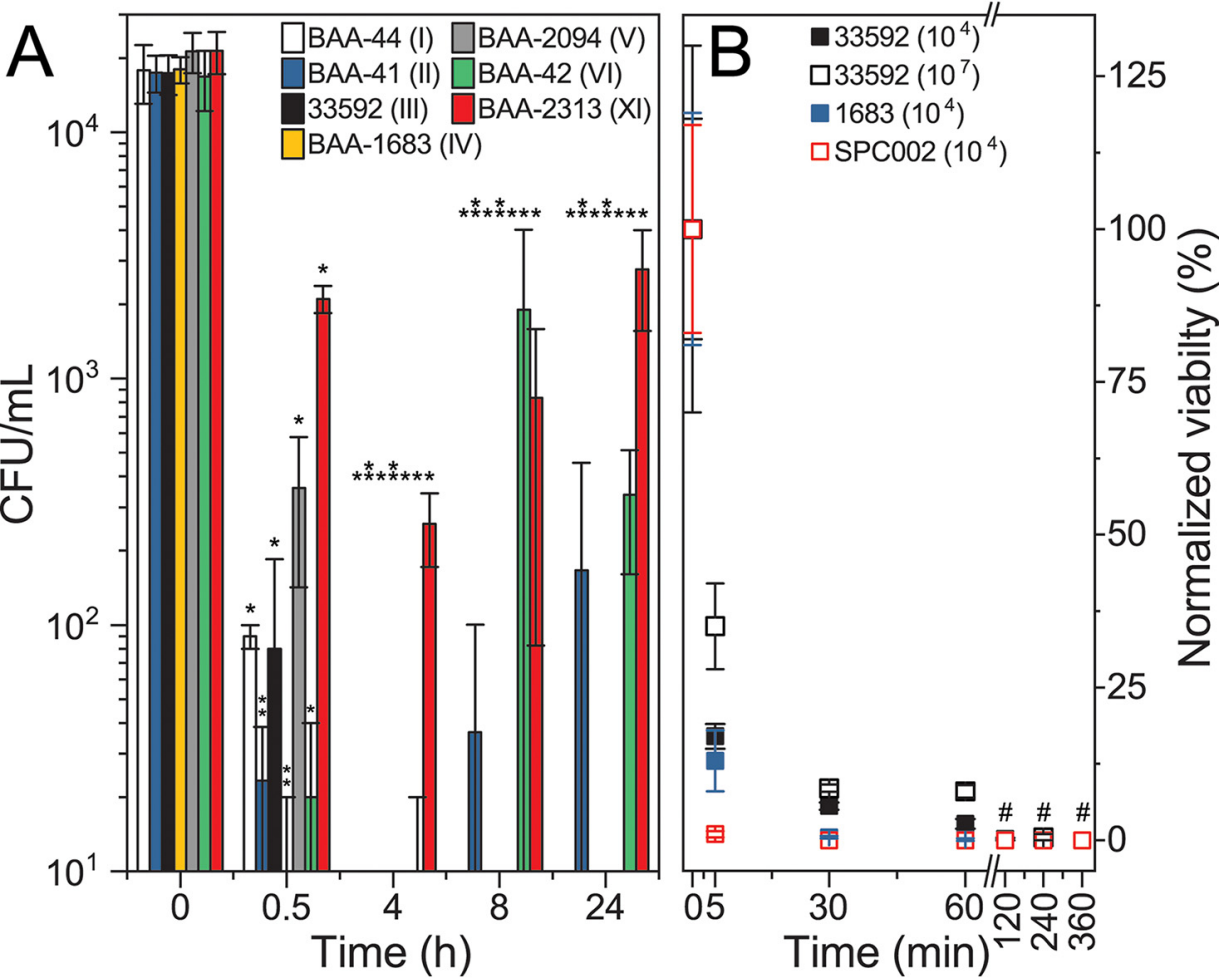
(A) Time-dependent changes to the viability of a panel of 7 MRSA strains treated with STIP3-29. The mean counts of viable bacteria are reported in CFU/mL and the standard deviation is shown for three biological replicates. Statistically significant differences for each sample relative to pretreatment with STIP3-29 (time = 0 h) are indicated with asterisks (*, *P < *0.05; ** shown vertically, *P < *0.01) (B) The effect of STIP3-29 on a subset of bacteria, including MRSA ATCC 33592, MRSA ATCC 1688, and S. pseudintermedius SPC002. Viability was normalized to the starting cell count of each strain: for MRSA ATCC 33592, the experiment was performed at initial cell counts of 10^4^ and 10^7^ CFU/mL, whereas the initial counts for the other strains were on the order of 10^4^ CFU/mL (indicated in parentheses). The # indicates that the number of viable cells was below the detection limit of the assay (10 CFU/mL).

We next focused on characterization of STIP3-29, determining that it was bactericidal by measuring MRSA viability during two time course experiments. First, all 7 MRSA strains were treated with 12.5 μg/mL STIP3-29 (2× MIC) in liquid broth for defined time intervals before plating to recover viable cells on STIP-free agar. STIP3-29 treatment resulted in the recovery of less than 1% of MRSA cells in 4 h (Fig. 3). After 24 h, MRSA ATCC BAA41, BAA-42, and BAA-2313 appeared to partially resist but not escape treatment. This observation may be explained by strain-level differences in persister cells as previously reported for S. aureus treated with other antibiotics (27), but it differs from reports that S. aureus PSMα peptides can suppress persister cell development (28, 29). To better define the timescale of the bactericidal effect of STIP3-29, a second experiment with two MRSA isolates (ATCC 33592 and ATCC 1683) as well as S. pseudintermedius (SPC002; Fig. 3, inset) was performed, showing a significant decrease in viability of all three strains within 5 min of treatment (≤40%) and inability to recover viable bacteria after 2 h of treatment.

### STIPs are characterized by structural plasticity and a nonstereospecific target.

**FIG 4:**
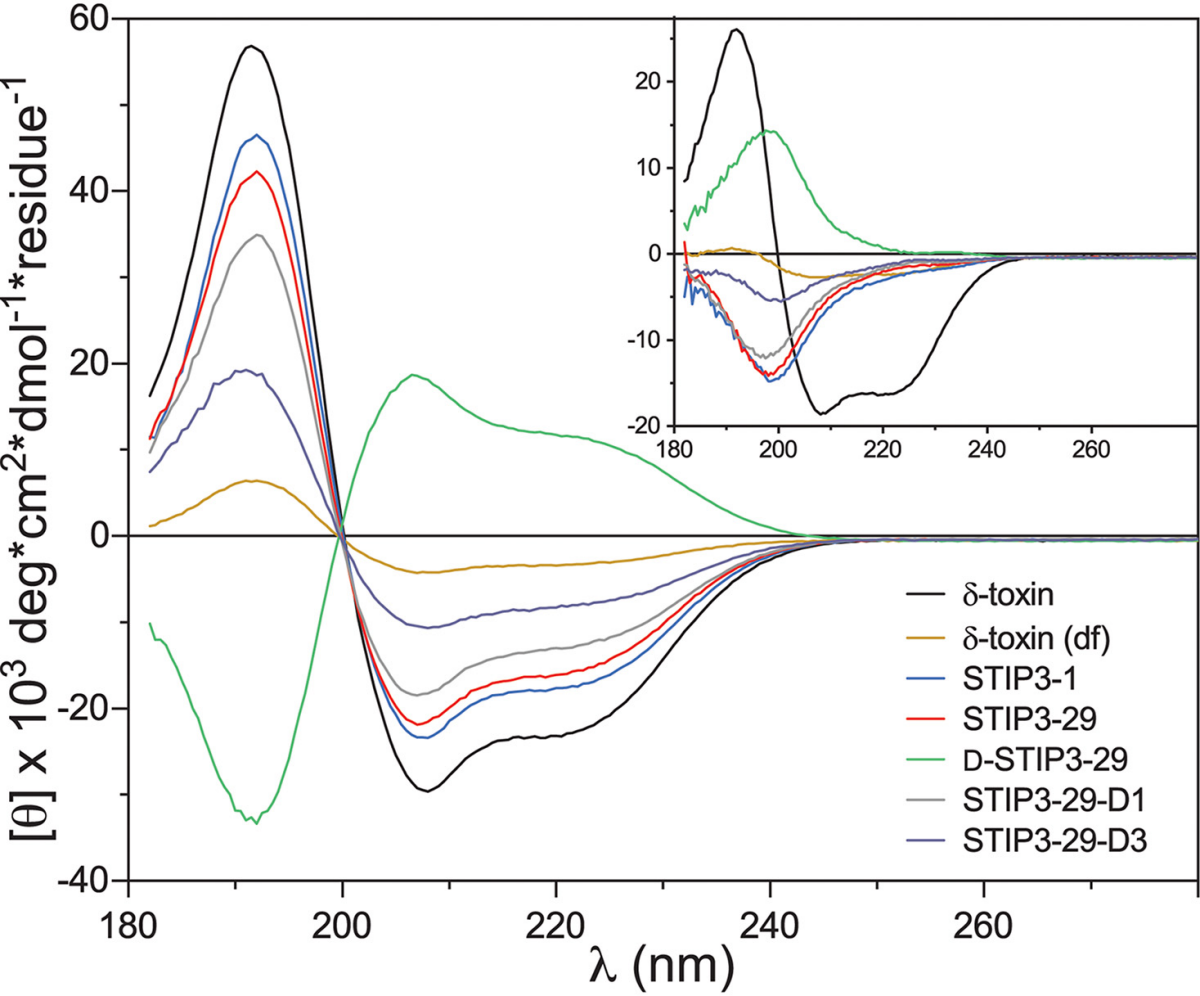
CD spectra of STIP3 variants and the *S. auricularis* δ-toxin in 50% TFE (vol/vol) and water (inset).

To investigate structural determinants of STIP activity, we measured peptide secondary structures using circular dichroism (CD) spectroscopy. The S. aureus δ-toxin and other PSMs are known to form α-helices, higher order oligomers, and to possess an ability to form amyloid fibers (30–32). STIP3-1 and its variants appear unstructured in water and α-helical in 50% trifluoroethanol (TFE; vol/vol) whereas the *S. auricularis* δ-toxin was α-helical in both solvents (Fig. 4; Table S5). Helicity has been reported as a major determinant of the δ-toxin binding to phospholipid bilayers (33), though the α-helical content of STIPs in TFE was not correlated with antibiotic activity.

To further test the relationship between peptide structure and activity, three additional STIP3-29 variants were studied: (i) an all d-enantiomer (d-STIP3-29) and (ii) a single (STIP3-29-D1) or (iii) three d-Lys residue-containing peptide (STIP3-29-D3) at position 11 or positions 7, 11, and 15, respectively (Table 1). The introduction of one or three d-Lys residues increased the MIC of the peptides by 4-fold or to the point of inactivation for STIP3-29-D3, which also showed limited α-helical content. The d-enantiomer matched the MIC of the l-enantiomer (6.25 μg/mL). Functional equivalence of l- and d-enantiomers has been observed for AMPs that are thought to function through the formation of membrane-spanning ion channels (34), and the cytotoxic effects of l- and d-PSMα3 are also indistinguishable from each other (35). Overall, the results suggest that structural plasticity is an important determinant of activity and implicate a nonstereospecific interaction with target cells.

### STIP3-29 is a cell-penetrating peptide.

**FIG 5:**
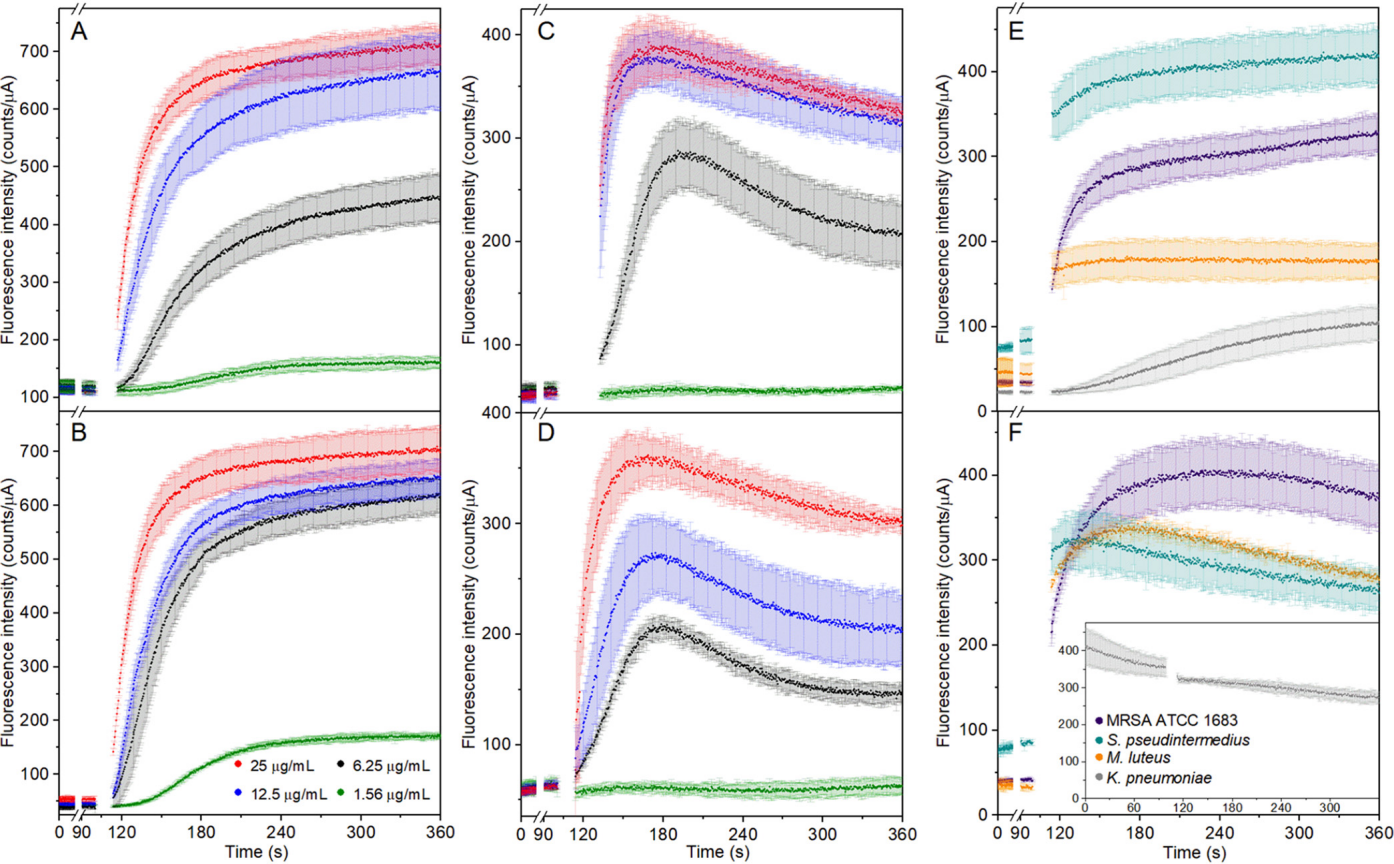
(A) Time-dependent changes to SYTOX Green fluorescence mixed with MRSA ATCC 33592 treated with STIP3-29 and (B) d-STIP3-29. (C) Time-dependent changes to DiSC_3_(5) fluorescence mixed with MRSA ATCC 33592 treated with STIP3-29 and (D) d-STIP3-29. (E) Time-dependent changes to SYTOX Green and (F) DiSC_3_(5) fluorescence mixed with MRSA ATCC BAA-1683, S. pseudintermedius (SPC002), M. luteus, and K. pneumoniae treated with 25 μg/mL STIP3-29. The concentrations of peptides used in panels A to D are listed and color coded in panel B.

We employed two indirect assays to assess the interaction of STIP3-29 and d-STIP3-29 with bacterial membranes. First, we measured changes to SYTOX Green fluorescence, a cell-impermeable DNA-binding dye, when MRSA ATCC 33592 was treated with peptide concentrations from 0.25- to 4-fold the MIC. Rapid and concentration-dependent increases in fluorescence were observed (Fig. 5A and B), consistent with STIP3-29 treatment providing the dye access to bind DNA. Next, we utilized DiSC_3_(5), a cationic dye that binds polarized membranes, to report on STIP-dependent changes. Again, we observed rapid dose-dependent increases in fluorescence (Fig. 5C and D), consistent with the release of self-quenched DiSC_3_(5) molecules from the bacterial membrane. The biphasic nature of the changes to fluorescence intensity, specifically the slow decrease observed after DiSC_3_(5) release from the bacterial membrane, is the result of photobleaching (Fig. S4). Rapid DISC_3_(5) release was also observed for three additional STIP-sensitive strains (Fig. 5E and F). In contrast, when a resistant bacterium, Klebsiella pneumoniae, was treated with STIP3-29, limited and slow SYTOX Green fluorescence was observed and no cell or peptide-specific changes to DiSC_3_(5) fluorescence were observed.

**FIG 6:**
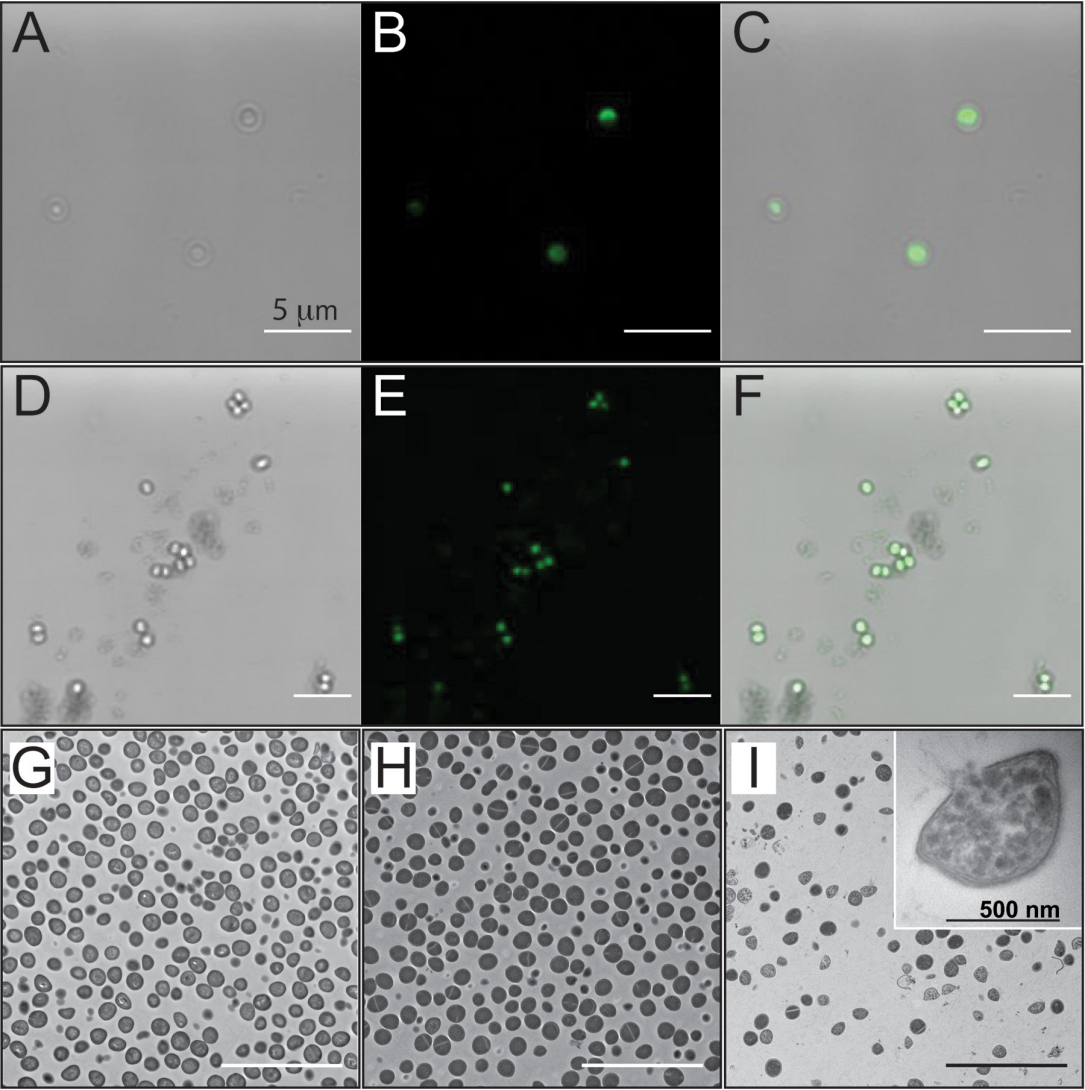
Microscopic images of MRSA ATCC 33592 treated with STIP3-29. Confocal images (83.2× magnification) show cells treated with 25 μg/mL FAM-l-STIP3-29 or FAM-d-STIP3-29 for 30 min, including bright field (A or D), fluorescent (B or E), and merged images (C or F). TEM images at 5,000× magnification show untreated cells (G) and cells treated with 25 μg/mL STIP3-29 for 30 min (H) and 4 h (I). Panel I inset shows a lysed MRSA cell at 60,000× magnification. The scale bar shown in each panel indicates 5 μm (labeled in panel A) with the exception of the scale bar inset in panel I.

To help interpret the results of our indirect fluorometric assays of bacterial membrane integrity, we evaluated the physical interaction of fluorescein (FAM)-labeled l- and d-STIP3-29 with bacterial cells. Specifically, MRSA ATCC 33592 was imaged after treatments with 50 μg/mL of each FAM-labeled peptide. Labeled peptides behaved similarly to unlabeled STIPs in that they maintained an ability to form α-helices and inhibited bacterial growth, albeit at higher concentrations (Table 1; Fig. S5). Images of MRSA obtained after 30 min of treatment with the FAM-labeled peptides clearly showed intracellular accumulation (Fig. 6A to F). While this observation differs from the localization of native PSMs and other AMPs like LL-37 at the surface of eukaryotic and bacterial membranes (36, 37), it mirrors a recent report of a PSMα-inspired AMP that inhibits E. coli (38). Taken together, the bulk and cell-level measurements suggest that STIP3-29 treatment can rapidly alter membrane integrity but does not cause rapid cell lysis. Cell penetration by these synthetic AMPs represents a significant deviation from the lytic mechanisms commonly assigned to the PSMs and δ-toxins. To test this hypothesis, we imaged STIP3-29 treated MRSA 33592 cells using transmission electron microscopy (TEM). Indeed, lysis occurred on a disparate timescale from STIP3-29 cell penetration. No peptide-induced lysis was observed after 30 min of treatment, though evidence was present after 4 h (Fig. 6G to I; Fig. S6). Thus, TEM imaging confirmed that the STIPs are cell-penetrating peptides that perturb membrane integrity without causing rapid membrane lysis or disintegration.

### STIP3-29 controls MRSA infection of an epithelial tissue model.

**FIG 7:**
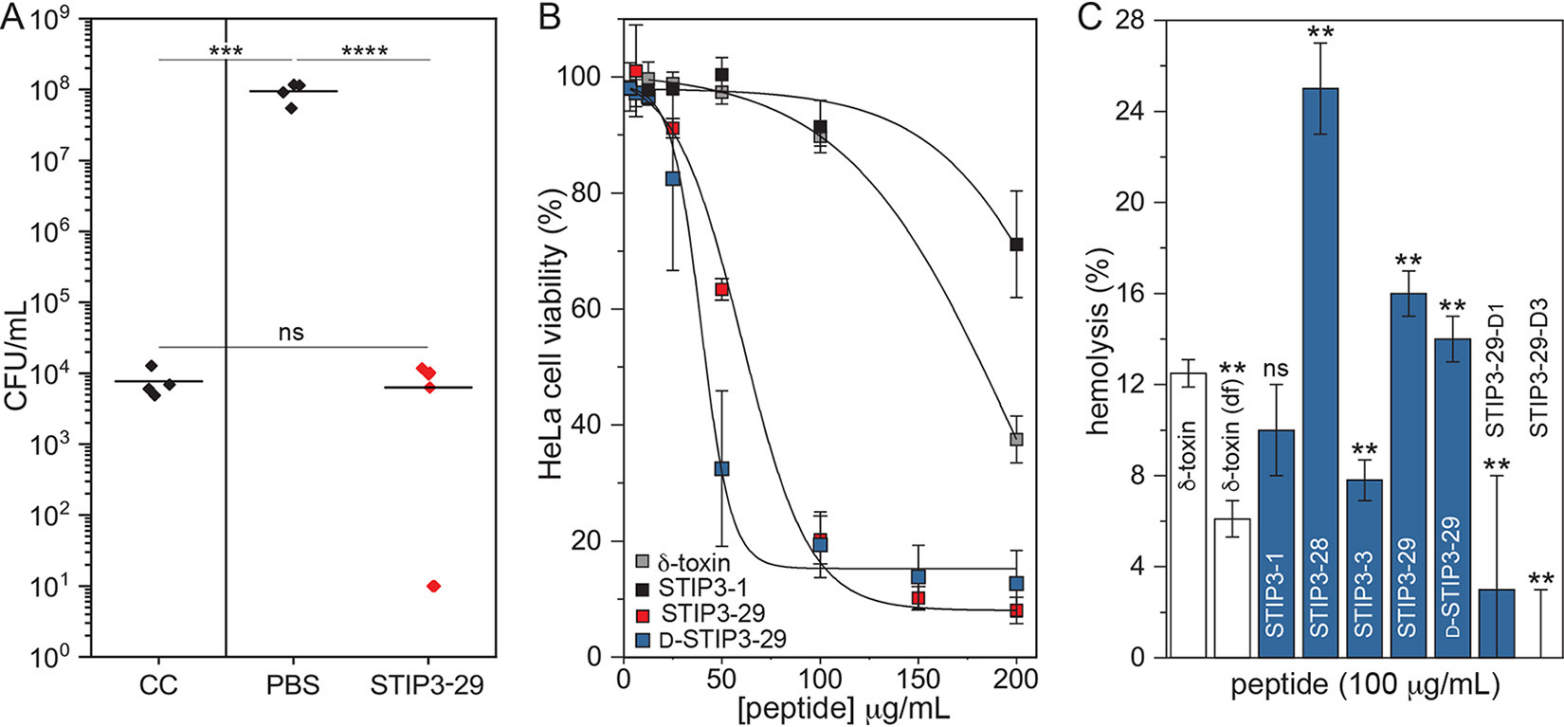
(A) Three-dimensional NHEK skin model of MRSA infection and AMP treatment. The concentration of MRSA cells recovered from colonized control tissue (CC) prior to treatment and tissues treated with PBS or 25 μg/mL STIP3-29 are shown. The vertical line between the CC group indicates that these counts were performed after 4 h of exposure to MRSA, whereas the PBS and STIP3-29 treatment groups were incubated for an additional 16 h. (B) Cytotoxicity of peptides measured against HeLa cells. Titrations of the δ-toxin, STIP3-1, STIP3-29, and d-STIP3-29 were performed between 3.13 and 200 μg/mL. (C) Bar graph showing relative hemolytic activity of *S. auricularis* δ-toxins and STIPs measured using sheep’s blood.

To begin to assess the potential utility of STIP3-29 as a topical antimicrobial agent, we used a three-dimensional (3D) epidermal skin model of infection. Tissues of normal, human-derived epidermal keratinocytes (NHEK) were colonized with MRSA 33592 using a 4 h exposure to 5 × 10^5^ CFU. Nonadherent cells were removed, leaving ∼7 × 10^3^ CFU (∼1%) of the MRSA colonized at the apical surface (Fig. 7A). Untreated MRSA populations expanded by 4-log units in a 16 h period, whereas the addition of 25 μg/mL STIP3-29 (4× MIC) prevented the expansion of the MRSA population. In two cases, we observed the ability of STIP3-29 to clear infection; however, the results were not universal. It is possible that differences in the behavior of biological MRSA replicates account for this ambiguity. For example, both the formation of AMP-resistant biofilms and protection through intracellular accumulation during the pretreatment window of infection may account for the discrepancy between replicates in this model. Despite the fact that the effect of STIP3-29 was not curative, the results demonstrate a capacity to control infection, making it a candidate for future optimization efforts as an antibiotic or antibiotic adjuvant.

Finally, we evaluated the cytotoxicity of several STIPs relative to their natural precursors. At the tissue level (data not shown), an indirect assay using a redox-sensitive dye was unable to differentiate STIP3-29-treated and untreated samples at 40-fold the MIC. These assays of stratified epithelia are unlikely to represent the apical surface. Thus, in addition to the NHEK tissues, the cytotoxicity of the *S. auricularis* δ-toxin, STIP3-1, STIP3-29, and the d-enantiomer was assayed using monolayers of HeLa cells. In this case, we observed a dose-dependent decrease in HeLa cell survival with 50% inhibitory concentration (IC_50_) values estimated at 230 ± 30, 200 ± 30, 56 ± 1, and 39 ± 3 μg/mL for the *S. auricularis* δ-toxin, STIP3-1, STIP3-29, and d-STIP3-29, respectively (Fig. 7B). For the l- and d-enantiomers of STIP3-29, maximum cytotoxicity was observed at a peptide concentration of ∼24-fold the bacterial MICs, and the ability of STIP3-29 to control MRSA infection was observed at a concentration with limited cytotoxicity. Although the cytotoxicity observed for STIP3-29 against HeLa cells was increased compared to that for the full-length δ-toxins, the hemolytic activity of the STIPs varied less drastically (Fig. 7C). Amidation of the C-terminus, which is present in STIP3-28 and STIP3-29, was correlated with an increase in hemolysis, whereas the inclusion of a single or three d-amino acids reduced the hemolytic activity. In the most extreme case, the level of hemolysis doubled relative to the formylated form of the δ-toxin. To provide a clinical context, all peptides tested, including the δ-toxin, can be classified to have a hemolytic index of “+” or as weakly hemolytic. In the future, histological observations of *ex vivo* and *in vivo* models will help to clarify the potential utility and toxicity of these AMPs.

### Conclusions.

Staphylococcal δ-toxin-inspired peptides are a potential reservoir of AMPs capable of killing MRSA and other opportunistic staphylococcal pathogens. By pairing a screen for AMPs with a pathogen-specific protease treatment, we identified a candidate from the products of a divergent δ-toxin sequence. Incremental improvements to STIP activity were achieved through simple rational design experiments, and remarkably, STIP3-29 demonstrated potential as a topical therapeutic agent for MRSA infections in a three-dimensional skin model. Preliminary biochemical analysis suggests that STIPs are capable of forming α-helices and act by rapidly translocating across membranes and accumulating within the cytoplasm of bacterial cells. How the STIPs ultimately kill the bacteria remains to be determined. As cell-penetrating peptides that alter membrane permeabilization, the STIPs may be useful as antibiotics and adjuvants that facilitate drug uptake. While we have demonstrated the ability of the STIPs to inhibit vegetative MRSA growth *in vitro* and within the context of a 3D tissue model, the effect of these peptides on staphylococcal biofilms remains to be explicitly determined. The STIPs are derived from a family known to modulate biofilm structure (13), which may result in synergistic activity with other clinically relevant and commonly used classes of antibiotics. Future efforts to define the mechanism of STIP action and robust structure-activity relationship studies should inform the design of more potent AMPs with promising therapeutic value.

## MATERIALS AND METHODS

### Reagents and peptides.

All reagents used in this study were of commercial origin. Synthetic peptides were purchased from GenScript (Piscataway, NJ); peptide purity of each ranged from 71% to 99%, and the value for each preparation has been provided in Tables S1, S4, and S5.

### Bacterial strains.

M. luteus ATCC 4698, S. aureus ATCC 29213, a panel of 7 MRSA (ATCC MP-2) strains, which includes ATCC BAA-41, BAA-42, BAA-44, BAA-1683, BAA-2094, BAA-2313, and 33592, and a series of additional bacterial isolates from human and veterinary cases were used in this study (Table S6). Bacterial strains were routinely grown in lysogeny broth (LB: 10 g/L tryptone, 5 g/L yeast extract, 10 g/L NaCl) at 30°C (M. luteus) or 37°C (*Staphylococcus* spp.) prior to use in specific experiments described below.

### Screening STIPs by spot-on-lawn assay.

Peptides (≥70% purity) were resuspended in sterile double distilled water (ddH_2_O) at a concentration of 10 mg/mL (gross weight), and M. luteus and S. aureus ATCC 29213 were used as indicator strains. To prepare test organisms, the optical density (OD) of overnight cultures was measured and the organisms were diluted to an initial OD of 0.05 (S. aureus) or 0.1 (M. luteus) in soft nutrient agar (0.4% wt/vol) at 50°C. Next, 3 mL of soft agar containing a test organism was overlaid on solid LB agar (1.5% wt/vol). A total of 50 μg of each peptide was spotted on the inoculated surface and plates were incubated for 16 to 48 h at 37 or 30°C.

### SspA protease treatment and peptide screen.

SspA protease was resuspended at 2 mg/mL in 100 mM HEPES (pH 7.8). Peptide samples (45 μL at 10 mg/mL) were mixed with SspA (5 μL at 2 mg/mL) and incubated at 37°C for a total of 5 h. The antimicrobial activities of the reaction mixtures were assayed by spotting 5 μL (∼45 μg) of each on either M. luteus or S. aureus test organisms as described above. Additional samples were flash frozen in liquid nitrogen and stored at −80°C prior to high-pressure liquid chromatography-mass spectrometry (HPLC/MS) analysis.

### HPLC/MS analysis.

HPLC/MS analyses were conducted to assess the proteolytic cleavage of STIPs by the SspA protease and catalogue peptides in the reaction mixtures. An Agilent Infinity II 1260 HPLC outfitted with a Phenomenex Lune C_5_ column (4.6 by 150 mm, 5 μm) was used to separate the peptide fragments using a binary solvent system consisting of solvent A (water, 0.05% formic acid) and solvent B (acetonitrile, 0.05% formic acid) operating at 0.7 mL/min. A 5-μL volume (∼4.5 μg) of each sample was injected at 10% solvent B, followed by a two-step gradient elution from 10% to 50% solvent B in 30 min, then 50% to 100% B in 10 min. The HPLC was coupled to an Advion CMS(L) single quadrupole mass detector through an electrospray ionization source, and ions were detected between 100 and 2,000 *m/z*. Low temperature and fragmentation settings were used: capillary temperature 135°C, capillary voltage 120 V, source voltage offset 20 V, source gas temperature 135°C, and electrospray ionization (ESI) voltage 3,500 V.

### Determination of MICs.

The MICs were measured for bioactive peptides identified from the initial screen. The purities of peptides used for MIC determinations are listed in Tables S4 and S5. Cation-adjusted Mueller-Hinton (MH) broth was used as the growth medium for MIC determination experiments. Bacterial cultures were diluted to an OD of 5 × 10^−4^ (∼10^5^ CFU/mL) in 200 μL MH broth mixed with peptides in a 2-fold dilution series arrayed in 96-well plates. Plates were incubated without shaking at 30 or 37°C depending on the organism. The MIC is defined as the lowest peptide concentration that resulted in no observable growth after 16 and 48 h for S. aureus and M. luteus, respectively.

### Measurement of bactericidal activity.

Time-dependent inhibition of pathogens was measured using standing bacterial cultures treated with 2× MIC. Overnight cultures were diluted to 10^4^ or 10^7^ CFU/mL in 2 mL of MH broth supplemented with the appropriate peptide concentration or sterile water as a control. Standing cultures were then incubated at 37°C and 100 μL aliquots were removed at specified time intervals. For the panel of 7 MRSA strains, treatment intervals of 30 min, 4 h, 8 h, and 24 h were used. For MRSA ATCC 33592, ATCC 1683, and S. pseudintermedius SPC002, shorter intervals included 5, 30, 60, 120, 240, and 360 min. The 100-μL aliquots were plated using serial dilutions as necessary on LB agar, and CFU were counted as a measure of bacterial viability after 24 h. To normalize cell viability data, surviving cells in CFU/mL were compared to the initial bacterial inoculum of each strain and then expressed as a percentage of that concentration. All experiments were performed in triplicate with values reported as the mean with standard deviations. Statistical differences in the observed viability of bacteria treated with STIP3-29 were analyzed using a paired two-tailed Student’s *t* test comparing the pretreatment (0 h) viability to those observed at posttreatment time points using Stata v17.0 software.

### Circular dichroism spectroscopy.

Peptides were dissolved in ultrapure water or 50% TFE (vol/vol) at a concentration of 0.2 mg/mL for spectroscopy. Spectra of samples in a 1 mm pathlength quartz cuvette were recorded from 180 to 260 nm at 0.5 nm steps using an Applied Photophysics Chirascan Plus CD spectrophotometer operating at room temperature.

### Evaluation of membrane permeability using SYTOX Green.

Fresh cultures of S. aureus (ATCC 33592 and 1683), S. pseudintermedius SPC002, M. luteus, and K. pneumoniae were grown to the log phase before they were harvested by centrifugation at 4,000 rpm and resuspended in phosphate-buffered saline (PBS; pH 7.4) supplemented with 12.5 μM SYTOX Green at an OD of 0.5 in a 10 mm pathlength quartz cuvette. Time-dependent changes in fluorescence were measured at room temperature using a Duetta spectrometer (Horiba Scientific) with excitation and emission wavelengths of 504 nm and 523 nm, respectively (5 nm bandpass).

### Evaluation of membrane depolarization using DISC_3_(5).

Fresh cultures of S. aureus (ATCC 33592 and 1683), S. pseudintermedius SPC002, M. luteus, and Klebsiella pneumoniae were grown to the log phase before they were harvested by centrifugation at 4,000 rpm and resuspended in PBS supplemented with 2.5 μM DISC_3_(5) at an OD of 0.5 in a quartz cuvette. Time-dependent changes in DISC_3_(5) fluorescence were measured using a Duetta spectrometer (Horiba Scientific). Fluorescence was recorded at room temperature using excitation and emission wavelength of 650 nm and 675 nm, respectively (5 nm bandpass).

### Confocal microscopy.

Images of N-terminally FAM-labeled peptides and MRSA ATCC 33592 cells were obtained using a Leica TCS SP5 confocal laser scanning microscope. The S. aureus cells were resuspended in PBS at an OD of 0.5, treated with 50 μg/mL peptide, and incubated at 37°C for 30 min. Cells were washed three times with PBS to reduce background fluorescence intensity before being mounted on glass slides. Images were capture at a total magnification of 83.2× using a 64× objective and oil immersion (1.4×). Fluorescence was excited using a 488-nm laser, and emitted light was collected between 500 and 606 nm. Images were produced using ImageJ 1.53c software (39).

### Transmission electron microscopy.

Samples for TEM imaging were prepared by diluting MRSA 33592 cultures to an OD of 0.5 in PBS before the cell suspension was treated with 25 μg/mL STIP3-29. Treated and untreated cells were incubated at 37°C. Treated samples were prepared from imaging after 30 min and 4 h of treatment. An untreated control sample was prepared after a 4 h incubation period. Cells from the distinct time points or treatments were harvested by centrifugation, fixed for 4 h using 2% glutaraldehyde, postfixed for 1 h with 1% OsO_4_, dehydrated with a graded ethanol series, embedded in Epon, and en bloc stained with saturated uranyl acetate in 70% ethanol prior to sectioning. Imaging was performed using a Hitachi HT7700 instrument.

### Three-dimensional tissue infection and treatment.

3D EpiDerm skin tissues (MatTek EPI-200-AFAB; antifungal and antibiotic-free; 9 mm) contained within a plastic insert lined with a permeable 0.4 μm membrane were transferred to 12-well plates containing antibiotic assay medium (600 μL/well). A fresh MRSA ATCC 33592 culture in the log phase of growth was centrifuged at 10,000 rpm for 5 min to harvest bacterial cells before resuspension in sterile PBS to an OD of 5 × 10^6^ CFU/mL. Tissues were incubated with 100 μL of the bacterial cell suspension (5 × 10^5^ CFU) for 4 h at 37°C in a humidified 5% CO_2_ environment. After 4 h, the bacterial cell suspension was removed and the apical surface of each tissue was washed 3 times with PBS to remove nonadherent bacterial cells. Next, the colonized skin tissues were treated with a 100 μL volume of STIP3-29 at 25 μg/mL. An equal volume of sterile PBS was used for untreated control experiments. Tissues were then incubated at 37°C in a humidified 5% CO_2_ environment for 16 h. To measure MRSA colonization, liquid treatments were removed from the apical surface, and the NHEK tissues were peeled from the 9-mm plastic insert using sterile forceps. The tissues were placed in a 400 μL sterile aliquot of PBS before disruption by vortexing for 1 to 2 min to detach and recover bacterial cells. Samples were then concentrated by centrifugation, and 100-μL resuspensions of bacterial cells were used for plating serial 10-fold dilutions of the samples on LB agar plates that were incubated overnight at 37°C. The number of MRSA colonies on each plate was counted and an average CFU/mL was determined for treated and untreated samples. This process was also performed after the 4 h colonization period to confirm that a population of adherent cells had been established in the skin model. The statistical differences in colonization were evaluated using an unpaired two-tailed Student’s *t* test to compare pretreated (control colonization; CC), untreated control (PBS), and STIP3-29 treated groups.

### Peptide cytotoxicity assays.

Cytotoxicity was measured using the 3D EpiDerm skin tissues and HeLa (ATCC CCL-2) cells using a fluorescent reporter of cell viability, alamarBlue (ThermoFisher Scientific). 3D EpiDerm skin tissues (MatTek EPI-200-AFAB) were transferred to a 12-well plate containing 600 μL of AFAB-assay medium supplemented with 10% vol/vol alamarBlue. STIP3-29 (250 μg/mL; 40-fold MIC) or PBS was added to the apical surface of the tissues and incubated at 37°C in a humidified 5% CO_2_ environment for 24 h. HeLa cells were seeded in a 96-well plate at 2 × 10^4^ cells/per well and incubated overnight at 37°C in a humidified 5% CO_2_ environment to prepare wells at ∼50% confluence for treatments after spent Dulbecco’s modified Eagle’s medium (DMEM) was removed and cells were washed with PBS. Assays were then performed using phenol red-free DMEM supplement with 9% vol/vol alamarBlue and peptide concentrations varying from 3.13 to 200 μg/mL. After a 24 h incubation period, alamarBlue fluorescence emission was measured at 590 nm upon excitation at 560 nm using Varioskan Lux plate reader (Thermo Scientific). Fluorescence intensities of treated cultures were normalized to measurement made for untreated control samples. Experimental data sets were fit to nonlinear dose-response curves to determine IC_50_ values using Origin 2019.

### Hemolytic activity assay.

Thirty-five percent defibrinated sheep’s blood (vol/vol) was centrifuged for 5 min at 4,000 rpm to harvest red blood cells. The cells were washed three times with PBS and harvested by centrifugation prior to dilution to 15% (vol/vol) in PBS in order to evaluate peptide-mediated hemolysis. The assay was performed in 96-well microtiter plates using peptides at 100 μg/mL in a 200-μL volume. Sterile water was used as a negative control and Triton X-100 (0.1% vol/vol) was used as a positive control for complete lysis. After 1 h of incubation, the 96-well microtiter plates were centrifuged at 4,500 rpm for 10 min and a 150 μL aliquot of the supernatant from each sample was carefully transferred to a new 96-well plate. The absorbance of the supernatant was then measured at 540 nm using a Varioskan Lux plate reader (Thermo Scientific). The hemolytic activity is reported relative to complete lysis (%) obtained using Triton X-100. Experiments were performed in triplicate, and statistical differences in hemolysis were measured using an unpaired two-tailed Student’s *t* test to compare the STIPs and deformylated δ-toxin to the formylated δ-toxin.
