# Supplementary material for: Cell-Penetrating Antimicrobial Peptides Derived from an Atypical Staphylococcal δ-Toxin
Source: Microbiol Spectr. 2021 Dec 22;9(3):e01584-21. doi: 10.1128/spectrum.01584-21 (PMC8694143; doi:10.1128/spectrum.01584-21)
Supplement: SUPPLEMENTAL FILE 1 — Supplemental material. Download SPECTRUM01584-21_Supp_1_seq9.pdf, PDF file, 0.7 MB [file spectrum01584-21_supp_1_seq9.pdf]

Supplementary Material for

Cell-penetrating antimicrobial peptides derived from an atypical staphylococcal δ-toxin

Kathyana Deeyagahage[1] and Antonio Ruzzini[1,2,*]

[1]Department of Veterinary Microbiology and [2]Department Biochemistry, Microbiology and Immunology, University of Saskatchewan, SK, Canada

*address correspondence to antonio.ruzzini@usask.ca

Table of Contents

Table S1. Sequences, purity and characteristics of peptides screened in this study S2
Figure S1. Spot-on-lawn to screen STIPs and SspA-treated STIPs against *M. luteus* S2
Figure S2. Spot-on-lawn assay of possible SspA cleavage products of STIP3 S2
Figure S3. Sequence alignment of 51 unique δ-toxins S3
Table S2. Summary of STIP fragments resulting from SspA treatment S4
Table S3. Summary of STIP3-1 and δ-toxin fragments resulting from SspA treatment S5
Table S4. Summary of MICs observed for STIP3-1 alanine variants in liquid broth S5
Table S5. Sequences, purity, and MICs of rationally-designed STIP3 variants S6
Table S6. MICs of STIP3-29 for *Staphylococcus* spp. and Gram-negative bacteria S7
Figure S4. Time-dependent change to $DISC_3(5)$ fluorescence in PBS S7
Figure S5. CD spectra of FAM-labeled peptides S8
Figure S6. TEM images of STIP3-29 treated MRSA 33592 at 5 and 20K magnification S8
Figure S7. TEM images of STIP3-29 treated MRSA 33592 at 40 and 60K magnification S9

Table S1. Sequences, purity, characteristics and source organisms of peptides screened for antibiotic activity.

Name	Peptide Sequence[a]	Purity	MW	pI	Source Organism
Δ1-2PSMα2	(MS)IIAGIIKFIKGLIEKFTGK	72.1%	2089.6 Da	10.00	*S. aureus*
STIP1	(MAAD)IISTISDLVKWIIDTVNKFTK	85.1%	2434.9 Da	8.50	*S. aureus*
STIP2	(MAQD)IISTIGDLVKWIIDTVNKFTK	89.5%	2404.9 Da	8.50	*S. aureus*
STIP3	(MTKD)IVETVGGLVKWILDTVKKFA	86.7%	2216.7 Da	8.50	*S. auricularis*
STIP4	(MAAD)IISTIVEFVKLIAETIAKFMK	94.4%	2395.0 Da	8.50	*S. cornubiensis*
STIP5	(MAAN)IISTIGDLVKWIIDTVNKFKK	91.2%	2431.9 Da	8.53	*S. epidermidis*
STIP6	(MAGD)IIGTINDLIKWIADTVEKYKK	82.8%	2461.9 Da	8.38	*S. massiliensis*
STIP7	(MAAD)IISTIGDLIKWIIDTVKKFKK(K)	70.8%	2460.0 Da	9.83	*S. saccharolyticus*

a – amino acids in parentheses were omitted from the peptides in the screen; SspA cleavage sites are shown in red

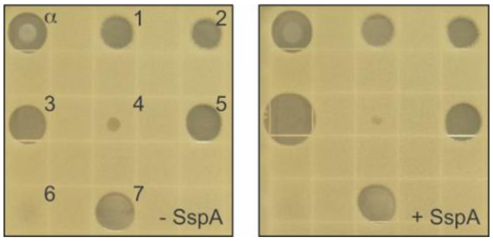

Figure S1. Results of a spot-on-lawn assay showing the inhibition of *M. luteus* ATCC 4698 by untreated or SspA-treated STIPs and PSMα1Δ1-2 (α: IIAIIKFIKGLIEKFTGK). Labels for the relative position of each spot appear on the left panel, and the order was reproduced on the right. A total of 50 μg of untreated and 45 μg of SspA-treated peptides were used in the assay. The opaque spots visible for the ΔPSMα1Δ1-2 are the result of precipitated peptide, which is less prominent after SspA treatment and not visible for the water-soluble STIPs.

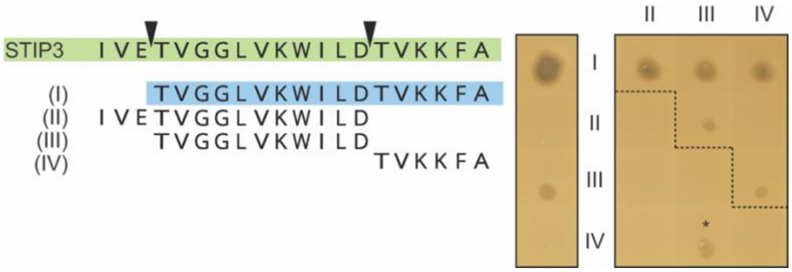

Figure S2. Antibiotic activity of four possible SspA-mediated STIP3 cleavage products. Arrows indicate expected sites of proteolysis; both complete and incomplete SspA cleavage products were evaluated (left panel). The results of a spot-on-lawn assays showing inhibition of *S. aureus* by single reaction products (I-IV) and pairwise combinations thereof (above the dashed line; right panel). The asterisk shows relatively weak activity of a four-peptide mixture.

Sequence	Alignment (positions 1–26)
WP_145444199.1	MTQDIIETVTKFVKLIAETVKKFTK
*STIP4 (WP_159454536.1)	MAADIISTIVEFVKLIAETIAKFMK
WP_142381183.1	MVADIVGTIVEFVKLIAETVKKFTK
WP_041612998.1	MAGDIVGTIGDFVKLIIETVNKFTNK
WP_047132799.1	MAGDIVGTIGDFVKLIIETVNKFTKK
WP_105994213.1	MAGDIVGTIGEFVKLIIETVKKFTQK
WP_182673220.1	MVGDIVGTIGEFVKLIIETVQKFTQK
WP_158256887.1	MAGDIVGTIGEFIKLIIETVQKFTQK
WP_158257062.1	MAGDIVGTIGEFVKLIIETVQKFTQK
VEG63905.1	MTGNIVDTVKSFVNLILDTVKKYAK
WP_142382114.1	MAGDIMNTINGLIKWIIDTVEKYKK
*STIP6 (WP_009384249.1)	MAGDIIGTINDLIKWIADTVEKYKK
WP_100223791.1	MAGDIMGTITDLIKWIAETVEKYKK
WP_154835904.1	MAADIISTIGDLVKLIINTVKNSKNN
PXX83671.1	MAADIISTISDLVKLIINTVKKFQK
WP_123186446.1	MAGDIISTIGDLVKLIINTVKKFQK
WP_152373221.1	MTADIISTIGDLVKLIINTVKKFQK
WP_015364840.1	MAADIISTIGDLVKLIINTVKKFQK
*STIP3 (WP_103170786.1)	MTKDIVETVGGLVKWILDTVKKFA
WP_159463944.1	MTADIISTIGGFVKWILDTVKK
WP_050812696.1	MAADIISTIGDFVKWILDTVKKFTK
WP_015364841.1	MTADIISTIGDFVKWILDTVKKFTK
WP_154835928.1	MTADIISTIGDSVKWILDTVKKFAK
WP_072291871.1	MTADIISTIGDFVKWILDTVKKFAK
AHY24175.1	MAADIISTIGDLVKWIIDTVNKFKI
*STIP5 (WP_080009574.1)	MAANIISTIGDLVKWIIDTVNKFKK
WP_194374686.1	MAGDIISTIGDLVKWIIDTVNKFKK
WP_002464791.1	MAADIISTIGDLVKWIIDTVNKFSK
WP_002494082.1	MAADIISTIGDLVKWIIDTVNKFKK
WP_001277673.1	MAQDIISTIGDLVKWIIDTVNKFN
*STIP1 (BBG11466.1)	MAQDIISTISDLVKWIIDTVNKFTKK
NKO69276.1	MAQDIISTISDLVKWIIDTVNKFTKN
WP_094970105.1	MAQDIISTIGDLVKWIIDTVNKFTKE
WP_031863708.1	MAQDIISTIGDLVKWIIDTVNKFIKN
WP_049313024.1	MAQDIISTIGDLVKWIIDTVNKFTKN
WP_142240992.1	MDRDIISTIGDLVKWIIDTVNKFTKK
WP_147697316.1	MTQDIISTIGDLVKWIIDTVNKFTKK
WP_053876829.1	MPQDIISTIGDLVKWIIDTVNKFTKK
WP_140835818.1	MAQDIISTIGDLVKWIIDTVSKFTKK
WP_076679790.1	MAQDIISTIGDLVKWIVDTVNKFTKK
WP_117224220.1	MAQDIISTIGDIVKWIIDTVNKFTKK
WP_188363454.1	MAQDIISTIGELVKWIIDTVNKFTKK
WP_151095789.1	MAQVIISTIGDLVKWIIDTVNKFTKK
QOA82756.1	MAQGIISTIGDLVKWIIDTVNKFTKK
*STIP2 (AAF43204.1)	MAQDIISTIGDLVKWIIDTVNKFTKK
WP_049307263.1	MAADIVSTISDFIKWIIDTIKKFKK
WP_119520274.1	MASDIVSTISDFIKWIIDTIKKFKK
WP_145388191.1	MAADIVSTIGDLVKWIIETVKKFKK
WP_100209060.1	MAADIVSTIGDFIKWIIETVKKFKK
*STIP7 (WP_130548313.1)	MAADIISTIGDLIKWIIDTVKKFKKK
WP_115312980.1	MAADIISTIGDLIKWIIDTVKKFKKN
WP_080981464.1	MASDIISTISDLIKWIIDTVKKFKK

Figure S3. Multiple sequence alignment of 52 unique δ-toxin sequences. Acidic amino acids are highlighted in red. The STIPs are indicated with an asterisk and named. Two additional δ-toxin sequences encoded by *S. condimenti* (VEG63905.1) and *S. pettenkoferi* (WP_145444199.1) show acid amino acid spacing that is similar to STIP3, which is based on the *S. auricularis* δ-toxin sequence (WP_103170786.1).

Table S2. Summary of STIP fragments resulting from SspA protease treatment observed by LC/MS

Name	Peptides & detected fragments	Molecular ions (*m/z*) & identity observed	theoretical	
STIP1	IISTISDLVKWIIDTVNKFTK	1218.9	1218.4	$[M+2H]^{2+}$
		813.1	812.6	$[M+3H]^{3+}$
	IISTISD	748.8	748.8	$[M+H]^{+}$
	LVKWIIDTVNKFTK	570.1	569.3	$[M+3H]^{3+}$
	TVNKFTK	419.4	419.5	$[M+2H]^{2+}$
	LVKWIID	444.0	444.0	$[M+2H]^{2+}$
STIP2	IISTIGDLVKWIIDTVNKFTK	1204.1	1203.4	$[M+2H]^{2+}$
		802.9	802.6	$[M+3H]^{3+}$
		602.3	602.2	$[M+4H]^{4+}$
	IISTIGD	718.7	718.8	$[M+H]^{+}$
	TVNKFTK	419.4	419.5	$[M+2H]^{2+}$
	LVKWIID	444.0	444.0	$[M+2H]^{2+}$
STIP3	IVETVGGLVKWILDTVKKFA	1110.0	1109.3	$[M+2H]^{2+}$
		740.3	739.9	$[M+3H]^{3+}$
	IVE	360.3	360.4	$[M+H]^{+}$
	TVGGLVKWILDTVKKFA	626.3	626.1	$[M+3H]^{3+}$
	TVKKFA	693.9	693.8	$[M+H]^{+}$
		347.3	347.4	$[M+2H]^{2+}$
	TVGGLVKWILD	601.3	601.2	$[M+2H]^{2+}$
STIP4	IISTIVEFVKLIAETIAKFMK	799.6	799.3	$[M+3H]^{3+}$
		599.8	599.7	$[M+4H]^{4+}$
	IISTIVE	774.7	774.9	$[M+H]^{+}$
	FVKLIAETIAKFMK	820.9	820.5	$[M+2H]^{2+}$
		410.4	410.8	$[M+4H]^{4+}$
	TIAKFMK	419.9	420.0	$[M+2H]^{2+}$
STIP5	IISTIGDLVKWIIDTVNKFKK	811.9	811.6	$[M+3H]^{3+}$
		609.1	609.0	$[M+4H]^{4+}$
	IISTIGD	718.7	718.8	$[M+H]^{+}$
	TVNKFKK	432.9	433.0	$[M+2H]^{2+}$
	LVKWIID	887.3	887.1	$[M+H]^{+}$
		444.0	444.0	$[M+2H]^{2+}$
STIP6	IIGTINDLIKWIADTVEKYKK	822.1	821.6	$[M+3H]^{3+}$
		616.7	616.5	$[M+4H]^{4+}$
	IIGTIND	745.8	745.8	$[M+H]^{+}$
	KYKK	566.3	566.7	$[M+H]^{+}$
		283.8	283.8	$[M+2H]^{2+}$
	LIKWIAD	430.0	430.0	$[M+2H]^{2+}$
	LIKWIADTVE	594.6	594.7	$[M+2H]^{2+}$
STIP7	IISTIGDLIKWIIDTVKKFKK	821.5	821.0	$[M+3H]^{3+}$
		616.3	616.0	$[M+4H]^{4+}$
	IISTIGDLIKWIID	801.0	801.0	$[M+2H]^{2+}$
	TVKKFKK	440.0	440.1	$[M+2H]^{2+}$

Table S3. Summary of STIP3-1 and δ-toxin fragments resulting from SspA protease treatment observed by LC/MS

Name	Peptides & detected fragments	Molecular ions (m/z) & identity observed	theoretical	
STIP3-1	TVGGLVKWILDTVKKFA	469.8	469.8160	$[M+4H]^{4+}$
	TVGGLVKWILD	601.2	601.2125	$[M+2H]^{2+}$
	TVKKFA	347.4	347.4195	$[M+2H]^{2+}$
δ-toxin[a]	*f*-MTKDIVETVGGLVKWILDTVKKFA	1361.8	1361.1	$[M+2]^{2+}$
δ-toxin (deformyl)	MTKDIVETVGGLVKWILDTVKKFA	674.2	674.0625	$[M+4H]^{4+}$
	TVGGLVKWILDTVKKFA	626.1	626.09	$[M+3H]^{3+}$
	TVGGLVKWILD	1201.9	1201.43	$[M+H]^{+}$
		601.2	601.215	$[M+2H]^{2+}$
	TVKKFA	347.3	347.425	$[M+2H]^{2+}$
	MTKDIVE	835.7	835.97	$[M+H]^{+}$
		418.3	418.485	$[M+2H]^{2+}$

a – no SspA cleavage was observed

Table S4. Summary of MICs observed for alanine substituted STIP3-1 experiment in liquid broth

ID	Peptide Sequence	Purity	MIC (μg/mL)[a] *M. luteus*	*S. aureus*
WT	TVGGLVKWILDTVKKFA		12.5	100
T1A	AVGGLVKWILDTVKKFA	97.1%	12.5	200
V2A	TAGGLVKWILDTVKKFA	97.8%	50	>
G3A	TVAGLVKWILDTVKKFA	83.1%	50	>[b]
G4A	TVGALVKWILDTVKKFA	98.4%	12.5	>[b]
L5A	TVGGAVKWILDTVKKFA	83.6%	100	>
V6A	TVGGLAKWILDTVKKFA	92.3%	50	>
K7A	TVGGLVAWILDTVKKFA	97.7%	200	>
W8A	TVGGLVKAILDTVKKFA	98.3%	100	>
I9A	TVGGLVKWALDTVKKFA	90.3%	200	>
L10A	TVGGLVKWIADTVKKFA	95.1%	200	>
D11A	TVGGLVKWILATVKKFA	87.5%	3.13	100
T12A	TVGGLVKWILDAVKKFA	98.5%	12.5	100
V13A	TVGGLVKWILDTAKKFA	90.4%	50	200
K14A	TVGGLVKWILDTVAKFA	93.4%	12.5	>[b]
K15A	TVGGLVKWILDTVKAFA	96.5%	25	>[b]
F16A	TVGGLVKWILDTVKKAA	95.1%	200	>

a – the use of > indicates an MIC >200 μg/mL; the maximum concentration tested

b – inhibition observed on an agar surface (see Figure 1)

Table S5. Sequences, purity, and MICs of rationally-designed STIP3 variants tested against a panel of *S. aureus*

ID	Sequence[b]	Purity (%)	α-helical content (%)	***S. aureus* ATCC strain identifier (MRSA SCC*mec* type)[a]** 29213	BAA 44 (I)	BAA 41 (II)	33592 (III)	BAA 1683 (IV)	BAA 2094 (V)	BAA 42 (VI)	BAA 2313 (XI)
δ-toxin	*f*-MTKDIVETVGGLVKWILDTVKKFA	97.5	88	>	>	>	>	>	>	>	>
δ-toxin (df)	MTKDIVETVGGLVKWILDTVKKFA	95.1	87	>	>	>	>	>	>	>	>
STIP3-1	TVGGLVKWILDTVKKFA	98.3	67	100	100	100	100	>	100	100	100
STIP3-2	TVGGLVKWILATVKKFA	95.3		100	100	100	50	50	50	50	50
STIP3-3	TVGGLVKWILKTVKKFA	91.2		25	25	25	25	50	25	50	50
STIP3-4	TVGGLVKWILNTVKKFA	99.9		100	50	50	50	100	50	50	100
STIP3-5	TVGGLVKWILDTVKKFI	83.4		50	50	25	25	25	25	12.5	25
STIP3-6	TVGGLVKWILDTVKKFW	95.5		12.5	25	12.5	12.5	12.5	12.5	12.5	12.5
STIP3-7	TVGGLVKWILDTVKKFK	84.4		>	>	>	>	>	>	>	>
STIP3-8	TVGVLVKWILDTVKKFA	83.7		100	100	100	100	>	100	100	100
STIP3-9	TVGGLVKWILDVVKKFA	88.1		25	25	35	25	25	25	25	25
STIP3-10	TVGGLVKWILDWVKKFA	93.2		100	200	100	100	>	100	25	50
STIP3-11	TVGGLVKWILDTFKKFA	88.9		50	100	50	50	50	100	50	100
STIP3-12	TVGGLVKWILAAVKKFA	95.3		25	25	25	25	25	50	25	25
STIP3-13	TVGGLVKWILKAVKKFA	88.0		25	25	12.5	25	25	25	25	25
STIP3-14	TVKGLVKWILNVVKKFA	96.0		25	25	25	12.5	25	50	25	12.5
STIP3-15	TVAGLVKWILATVKKFA	94.2		25	25	50	12.5	25	25	50	50
STIP3-16	TVKGLVKWILATVKKFA	93.7		100	100	50	50	>	100	50	100
STIP3-17	TVDGLVKWILATVKKFA	96.1		>	>	>	>	>	>	>	>
STIP3-18	TVKKLVKWILKTVKKFA	92.3		100	100	100	100	>	100	>	>
STIP3-19	TVKALVKWILKTVAKFA	95.2		50	25	50	25	25	25	50	25
STIP3-20	TVRALVKWILRTVAKFA	97.3		25	25	12.5	25	25	25	25	50
STIP3-21	TVKALVKWILKTVAKFW	96.3		25	25	25	25	12.5	12.5	25	12.5
STIP3-22	TVXALVXWILXTVAXFA	84.6		25	25	25	25	25	25	25	100
STIP3-23	TVRALVRWILRTVARFA	88.8		25	25	6.25	25	25	50	50	25
STIP3-24	TVGVLVKWILNVVAKFA	82.5		>	>	>	>	>	>	>	>
STIP3-25	TVKFLVKWILKWVAKFA	96.4		>	>	>	>	>	>	>	>
STIP3-26	TVKVLVKWILKVVAKFA	84.5		>	>	>	>	>	>	>	>
STIP3-27	TVGGLVKWILDTVKKFA-NH_2	96.3		50	25	25	50	25	25	25	50
STIP3-28	TVGGLVKWILATVKKFA-NH_2	97.4		25	12.5	12.5	6.25	12.5	6.25	12.5	12.5
STIP3-30	TVKGLVKWILNVVKKFA-NH_2	95.9		12.5	12.5	25	12.5	12.5	25	25	25
STIP3-31	TVKALVKWILKTVAKFW-NH_2	97.4		25	25	25	25	25	25	50	25
STIP3-29	TVGGLVKWILKTVKKFA-NH_2	95.1	61	12.5	6.25	6.25	6.25	6.25	6.25	6.25	6.25
D-STIP3-29	TVGGLVKWILKTVKKFA-NH_2	98.6	47	6.25	12.5	6.25	6.25	6.25	6.25	6.25	6.25
STIP3-29-D1	TVGGLVKWILKTVKKFA-NH_2	96.3	51	12.5			25				
STIP3-29-D3	TVGGLVKWILKTVKKFA-NH_2	97.0	32	>			>				

a – > symbol indicates that no inhibition was observed at the highest concentration of peptide tested: 100 μg/mL for the STIP3 variants; 200 μg/mL for the δ-toxins
b – amino acid substitutions and modifications (C-terminal amidation) are indicated in blue; X indicates L-ornithine; D-amino acids are indicated in green.

Table S6. MICs of STIP3-29 required to inhibit other staphylococci and Gram-negative bacteria

**Bacterium**	**MIC (μg/ml)**
S. aureus	
bovid isolate (RDM-1)[a]	6.25
S. pseudintermedius	
human isolates[b]	
SPC001 (MSSP)	3.13
SPC002 (MSSP)	3.13
SPC020 (MSSR)	3.13
canine isolates[c]	
MSSP42	3.13
MRSP16	3.13
MRSP24	3.13
S. auricularis[d]	1.56
S. epidermidis[d]	3.13
S. equorum[d]	3.13
S. scuri[d]	3.13
Klebsiella pneumoniae[a]	>200
Pasteurella multocida[e]	>100
Pseudomonas aeruginosa ATCC 27853	50
Escherichia coli DH10B	12.5
Mannheimia haemolytica ATCC 33396	6.25

a – clinical isolate, causative agent of mastitis in a dairy cow
b – clinical isolates causing zoonotic disease(1), methicillin sensitive (MSSP) and resistant (MSSR) strains indicated
c – clinical isolates, Rubin laboratory, Western College of Veterinary Medicine, University of Saskatchewan
d – isolate from the healthy quarter of a dairy cow
e – clinical isolate, involved in beef cattle bovine respiratory disease

(1) Somayaji, R.; Priyantha, M. A. R.; Rubin, J. E.; Church, D., Human infections due to *Staphylococcus pseudintermedius*, an emerging zoonosis of canine origin: report of 24 cases. *Diagnostic Microbiology and Infectious Disease* 2016, 85 (4), 471-476.

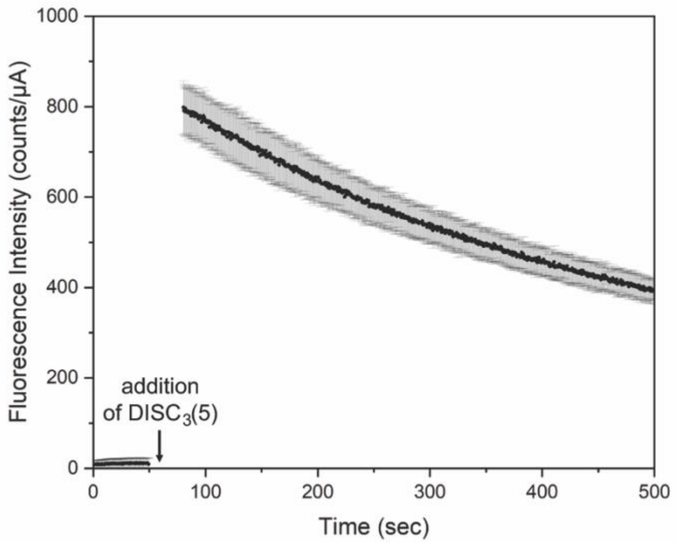

Figure S4. Time-dependent decay in $DISC_3(5)$ fluorescence at a concentration of 2.5 μM in PBS.

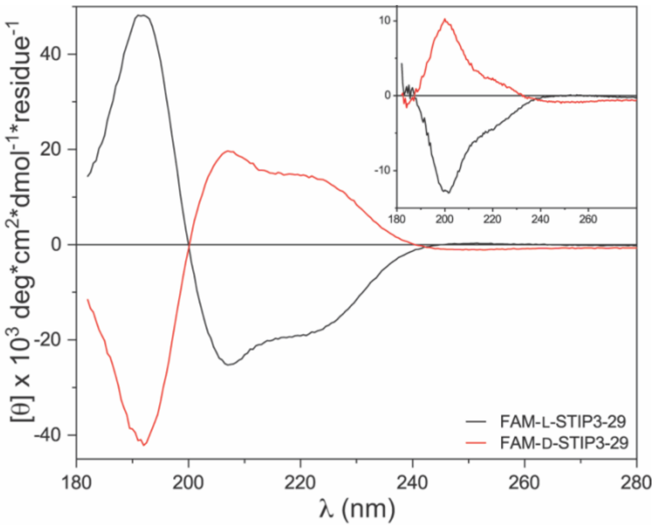

Figure S5. CD spectra of FAM-labeled STIP3-29 variants in 50% TFE (v/v) and water (inset).

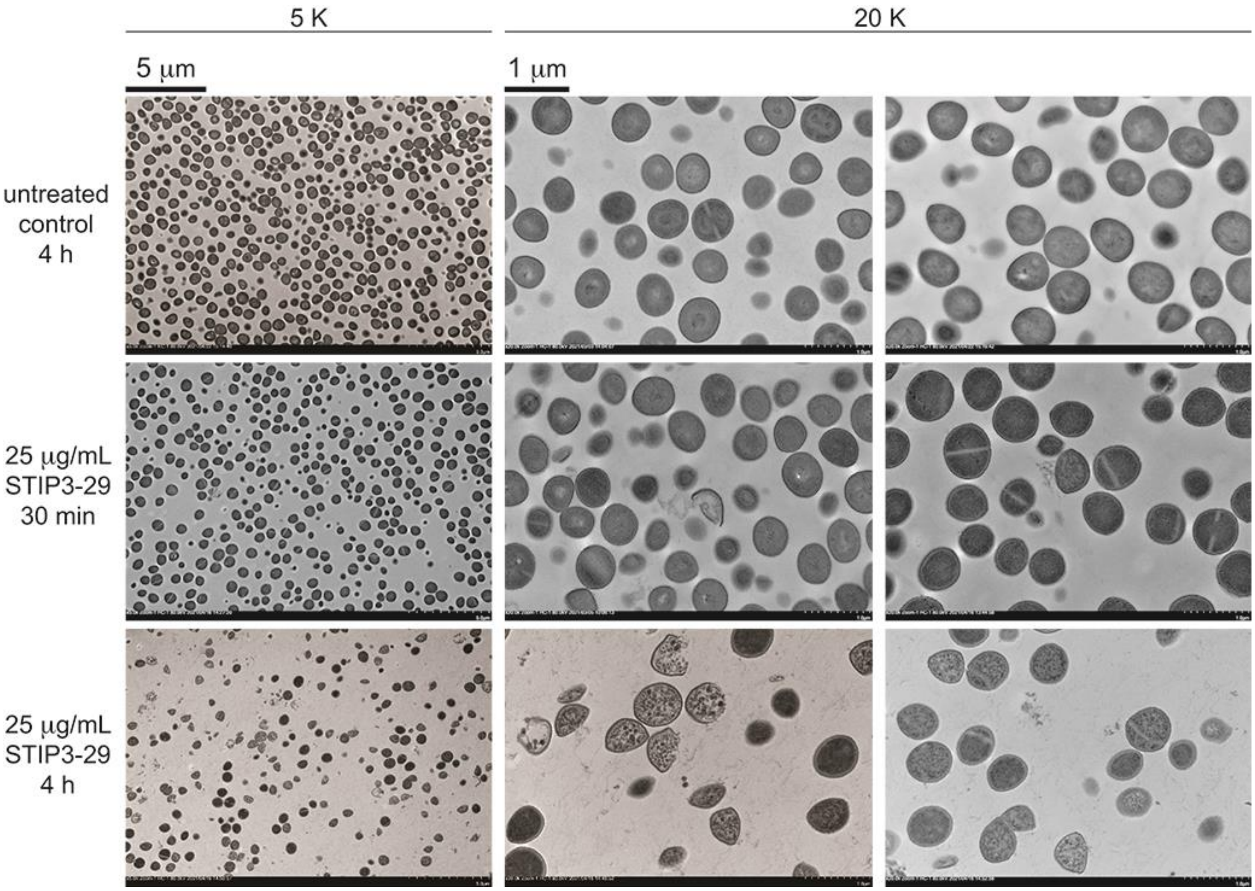

Figure S6. TEM images of MRSA 33592 with and without STIP3-29 treatment at 25 μg/mL. The top three panels show images of untreated cells incubated for 4 h. The middle three panels show cells after 30 minutes of treatment: little to no lysis was observed. The bottom three panels show MRSA after 4 h of STIP3-29 treatment. A significant reduction in cell number is observed with evidence of lysis and cell debris. Magnification and a scale bars are drawn at the top.

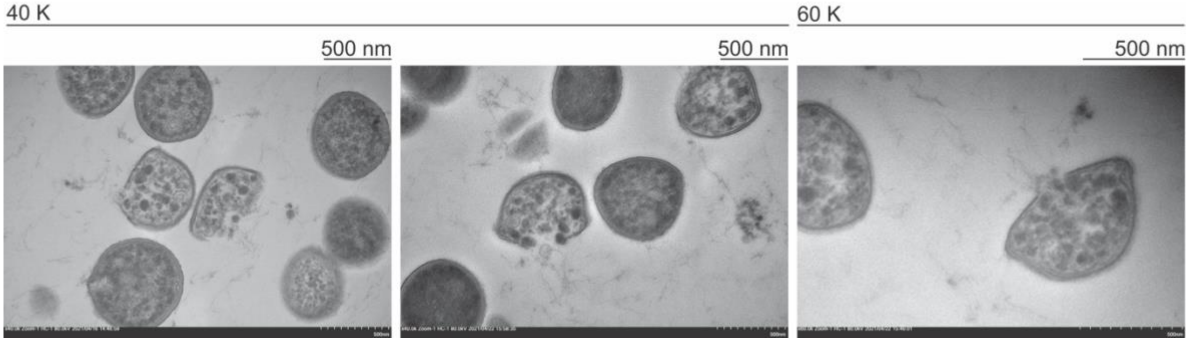

Figure S7. TEM images showing lysed MRSA 33592 cells treated with 25 μg/mL STIP3-29 for 4 h. The magnifications and scale bars are indicated at the top of the three panels.
